# Accuracy of a Digital Intraoral Scanner in Recording Occlusal Contact Points in Patients with Natural Dentition Compared to 8-µm Articulating Paper: A Cross-Sectional In-Vivo Study

**DOI:** 10.7759/cureus.109859

**Published:** 2026-05-29

**Authors:** Tolay Almahdi Altounsi, Mohammad Luai Morad, Mohammad Y Hajeer

**Affiliations:** 1 Department of Fixed Prosthodontics, Faculty of Dentistry, University of Damascus, Damascus, SYR; 2 Department of Orthodontics, Faculty of Dentistry, University of Damascus, Damascus, SYR

**Keywords:** articulating paper, diagnostic accuracy, digital dentistry, intraoral scanner, occlusal contacts

## Abstract

Background

Recent developments in digital dentistry have introduced intraoral scanners (IOSs) as potential alternatives to conventional methods for recording occlusal contact. However, clinical evidence comparing their diagnostic accuracy against the gold standard (8-μm articulating paper) remains limited, particularly regarding tooth-type-specific performance.

Objective

This cross-sectional comparative study evaluated the accuracy of the Cameo Elegant 3 intraoral scanner (IOS) for static occlusion contact detection compared to 8-μm articulating paper and determined whether tooth type (premolars vs. molars) influences contact point accuracy.

Materials and methods

Thirty-five patients (11 men, 24 women; aged 21-29 years) underwent occlusal assessment during a single visit. Occlusal contacts were marked with 8-μm Bausch articulating paper. The marked contact points were then immediately scanned using the Cameo Elegant 3 IOS. Digital scans of mandibular/maxillary arches and bite registration were obtained. Digital models were superimposed in Exocad software to identify matching and non-matching contact points (n=412 contacts across 140 posterior teeth). Point-level analysis was performed using confusion matrices and Cohen's kappa, while tooth-level data were analyzed using the Wilcoxon signed-rank test. Premolar-molar differences were assessed via chi-square/Fisher's exact tests, Mann-Whitney U, Spearman's correlation, and ROC curves using SPSS v27 (α=0.05).

Results

The scanner showed high sensitivity (90.4%, 95% CI: 87.4-93.0) but poor specificity (14.3%), with significant under-recording vs. articulating paper (p<0.001). Premolars outperformed molars (κ=0.115, p=0.002 vs. κ=-0.040, p=0.411), though overall agreement was negligible (κ=0.014).

Conclusion

The Cameo Elegant 3 intraoral scanner demonstrated high sensitivity but poor specificity for detecting static occlusal contact points compared with 8-μm articulating paper, with significant under-recording, particularly in the molar regions. While the scanner showed acceptable performance in premolars, the overall agreement was negligible. These findings indicate that IOSs should be used as adjunctive tools rather than being used as a standalone method for occlusal assessment. Combining digital scanning with conventional thin articulating paper remains essential for reliable clinical evaluation.

## Introduction

Accurate occlusal assessment is essential for the success of dental restorations, as it influences their functional stability and long-term clinical outcomes [1]. Occlusal forces play a direct role in determining the performance and longevity of dental restorations, and inappropriate occlusal loading may increase the risk of both mechanical and biological complications [1,2]. Consequently, a careful evaluation and management of occlusal relationships remain fundamental components of diagnosis and treatment planning in prosthodontics and restorative dentistry [3]. Traditionally, occlusal contacts have been assessed using articulating materials of varying thicknesses or thin aluminum shim stock (approximately 8 μm), which are widely regarded as reference methods in clinical practice. Human occlusal tactile sensitivity has been reported to be in the range of 8-10 μm, supporting the use of thin materials for detecting occlusal contacts. However, these conventional methods require repeated patient biting, are susceptible to saliva contamination, and provide only qualitative, rather than quantitative, information regarding occlusal force distribution [4]. With the rapid advancement of digital technologies, intraoral scanners (IOSs) have become integral to modern dental workflows, enabling the acquisition of three-dimensional virtual models without the need for conventional impression materials [5,6]. IOSs capture optical impressions and generate digital models that facilitate data storage, communication, clinical efficiency, and improved patient comfort [6]. Nevertheless, the performance and accuracy of IOS may vary depending on the device, software, operator experience, and clinical conditions [5,7]. Although IOSs have demonstrated clinically acceptable performance, evidence regarding their accuracy in detecting static occlusal contact points remains limited [7]. Furthermore, digital occlusal analysis systems are evaluated based on parameters such as sensitivity, specificity, trueness, and precision, emphasizing the importance of validating their clinical reliability [8]. Previous methods for occlusal assessment have largely relied on qualitative approaches, such as articulating paper, which depend on subjective interpretation [1,2].

These conventional techniques are also influenced by material properties and intraoral conditions, and they do not provide quantitative information regarding occlusal force magnitude or timing [8,9]. In contrast, digital technologies, including IOSs and computerized occlusal analysis systems, allow for enhanced visualization and analysis of occlusal contacts and are increasingly incorporated into clinical practice [5,7]. Virtual interocclusal records (VIRs) obtained using IOSs have been suggested to improve articulation accuracy by reducing human- and material-related errors within a digital workflow [10]. Variability in accuracy among different intraoral scanning systems has been reported, highlighting the need for careful evaluation when selecting and applying these technologies [7,9]. Although most existing studies have evaluated occlusion at a general or tooth level, with limited focus on detailed contact point analysis [3,8], this highlights the need for more precise and objective assessment methods. Digital IOSs such as 3Shape TRIOS®, Medit i-series, and iTero are widely used in clinical practice, and multiple studies have evaluated their accuracy in occlusal analysis [9,11]. However, research on cost-effective open systems like the Cameo Elegant 3 (Aidite Technology Co., Ltd., Qinhuangdao, China) remains limited, despite its compatibility with Exocad software and autoclavable tips. Therefore, the aim of this cross-sectional study was to evaluate the accuracy of the Cameo Elegant 3 intraoral scanner in recording static occlusal contact points, using 8-μm articulating paper as the reference standard. Additionally, as posterior molars exhibit different contact patterns and reduced scanner accuracy compared to premolars [12], differences in contact detection between premolars and molars were assessed. The null hypotheses of this study were that no significant difference exists in occlusal contact registration accuracy between the IOS and articulating paper, and that no significant difference exists in recorded occlusal contact points between premolars and molars.

## Materials and methods

Study design

This cross‑sectional study compared occlusal contact registration between 8-μm articulating paper and the Cameo Elegant 3 IOS, in patients with natural dentition, focusing on bilateral mandibular premolars and molars, at the Digital Dental Lab, Faculty of Dentistry, University of Damascus, between June 2025 and February 2026. This study was approved by the Biomedical Research Ethics Committee at the University of Damascus (No. DN-230426-782).

Calculation of the study sample

The study was designed to evaluate the agreement between occlusal contacts recorded using an IOS and conventional articulating paper. The required sample size was determined a priori using G*Power software (version 3.1.9.7, Heinrich Heine University, Düsseldorf, Germany) based on a two-tailed paired-design framework. In the absence of directly comparable in vivo studies using the same scanner system, a medium effect size (d=0.50) was selected according to Cohen’s conventional criteria to represent a clinically meaningful difference between the two recording methods. With the statistical power set at 80% (1−β=0.80) and the significance level at α=0.05, the minimum required sample size was calculated to be 35 participants.

Patient recruitment

After an assessment of 120 dental students from the Faculty of Dentistry at Damascus University, it was found that 70 students met the inclusion criteria. Each student received an information leaflet explaining the purpose and methods of the study. Each eligible student received an information leaflet explaining the purpose, procedures, potential risks, benefits, and their right to withdraw from the study at any time without consequences. Fifty students provided written informed consent to participate in the study. Afterward, a computer-generated sampling technique was employed to randomly choose 35 out of the 50 prospective patients. The inclusion criteria were as follows: Patients with intact bilateral posterior teeth (premolars and molars) in both jaws, with assessment limited to the mandibular posterior teeth, Class I skeletal occlusion according to Angle’s classification, and a preserved, reproducible occlusal relationship. The criteria for exclusion were as follows: Patients with temporomandibular joint or masticatory muscle disorders, large restorations or extensive caries on posterior teeth, bruxism, missing teeth in the posterior study area, periodontal or periapical diseases affecting the study’s teeth, or impacted third molars interfering with occlusion.

Assessing inter-occlusal contacts

The Gold Standard Method: The Traditional Method

**Figure 1:**
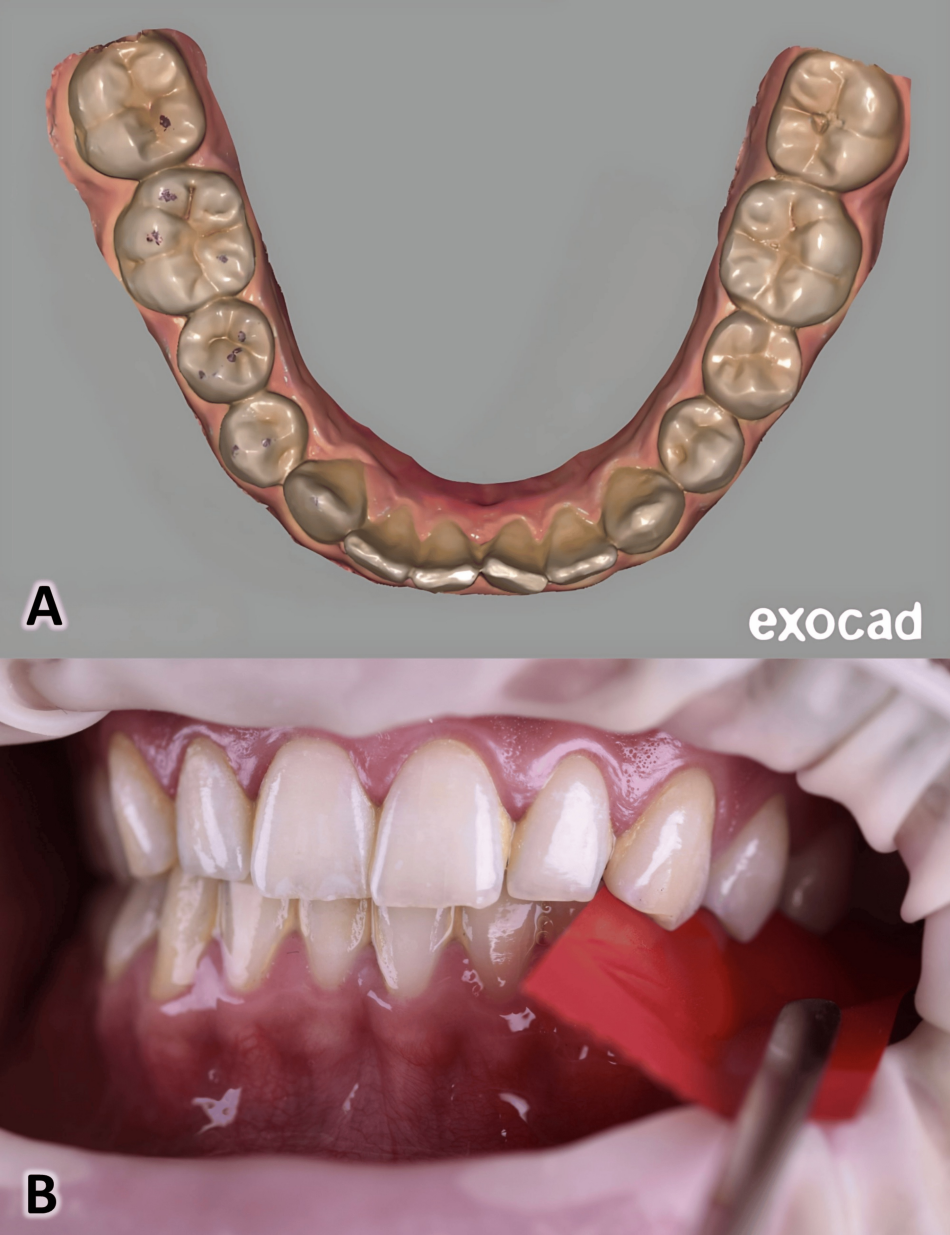
Maximum intercuspation with 8-μm articulating paper: A: Articulating paper markings on posterior teeth and B: Articulating paper application for occlusal contact point recording.

Prior to scanning, a lip retractor (OptraGate, Ivoclar Vivadent, Schaan, Liechtenstein) was used to enhance visibility, followed by thorough air-drying of the tooth surfaces. All patients were seated in an upright position with the head supported and stabilized. They were carefully instructed and guided to close into a reproducible natural maximum intercuspation (MIP) with moderate biting force. To ensure consistency of bite force and occlusal contact registration, 8-μm red and blue articulating paper (Arti-Fol, Dr. Jean Bausch GmbH & Co., Germany) was sequentially applied using forceps. A contact was considered valid only when it was consistently reproduced in two sequential markings with alternating colors. The occlusal surfaces with confirmed visible marks were immediately scanned using the Cameo Elegant 3 IOS to capture the marked locations. To minimize the influence of muscle fatigue and parafunctional habits, patients with a history of bruxism, temporomandibular disorders (TMD), or masticatory muscle pain were strictly excluded from the study according to the predefined exclusion criteria (Figure 1).

The Digital Method Using IOS

**Figure 2:**
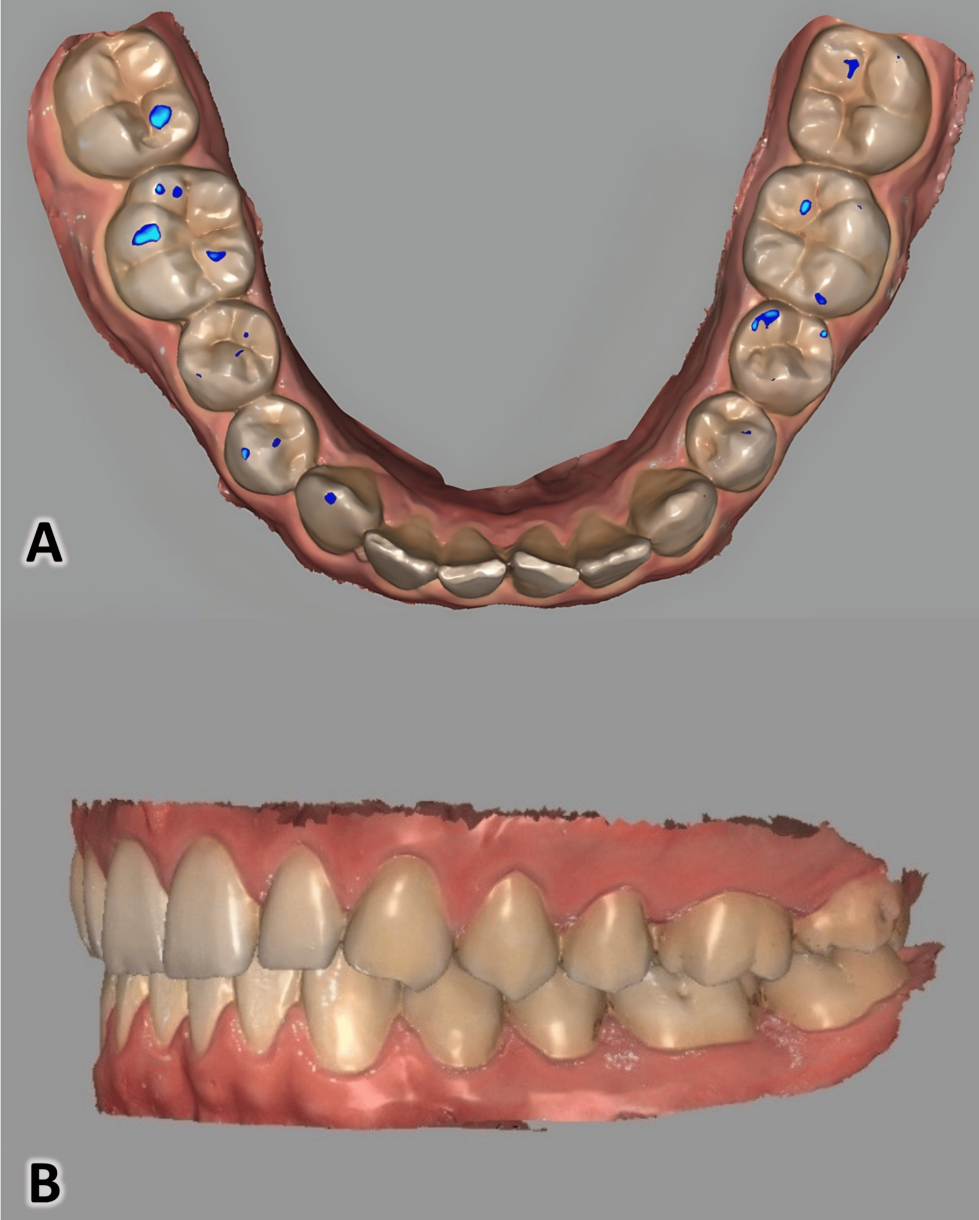
Maximum intercuspation captured by Cameo Elegant 3 scanner: A: Intraoral markings on posterior teeth and B: 3D visualization of intraoral scan generated by Cameo Elegant 3 scanner in Exocad DentalCAD™

Full arch scans were acquired using the Cameo Elegant 3 intraoral scanner following the manufacturer’s recommended protocol. The scanning sequence was the maxillary arch, followed by mandibular arch, then buccal surfaces in MIP for bite registration. The scanner’s tip was maintained perpendicular to the occlusal plane, and scanning was performed in a continuous motion from the canine to the first molar region. All scans were obtained under standard clinical lighting conditions and room temperature. Digital models were aligned in MIP using Exocad DentalCAD® (Exocad GmbH, Liechtenstein; Figure 2).

Occlusal analysis and model superimposition

**Figure 3:**
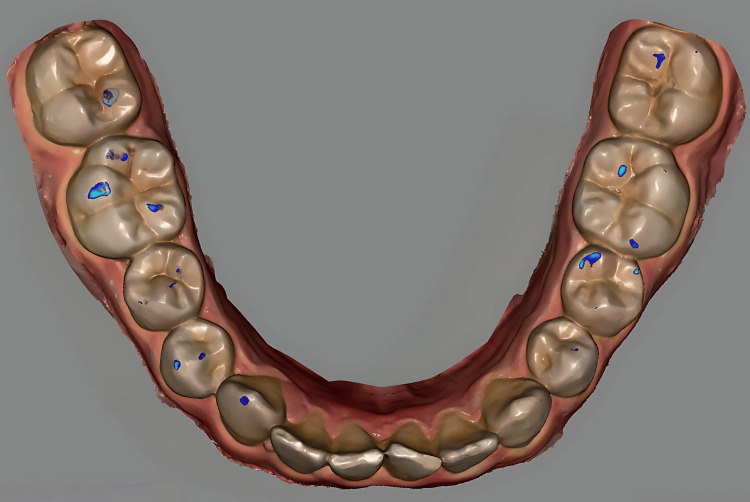
Superimposition of articulating paper markings (red/blue) with Exocad digital contacts on mandibular posterior teeth, showing matching (overlap) and non-matching regions.

The intraoral scans containing the visible articulating paper markings were superimposed with the digital occlusal contact maps in Exocad DentalCAD software (threshold ≤8-μm matching paper thickness; Figure 3). A single calibrated examiner then performed a visual point-by-point comparison to identify matching and non-matching contacts. Each contact point was classified as matching when the digital contact area overlapped with the location of the corresponding articulating paper mark and as non‑matching when the contact was recorded by only one of the two methods, representing either a false positive (FP) or a false negative (FN). Contact numbers per tooth were recorded in Microsoft Excel 365 (Microsoft Corp., Redmond, WA, USA). All clinical scans, digital model alignments, and point-by-point comparisons were performed by a single calibrated examiner to standardize the workflow and reduce inter-operator variability. Digital superimpositions were performed utilizing the automated best-fit alignment algorithms within Exocad software based on stable anatomic landmarks; however, a formal test-retest intra-examiner reliability analysis was not conducted.

Statistical analysis

Statistical analysis was performed using SPSS version 27 (IBM Corp., Armonk, NY, USA). Descriptive statistics (mean, standard deviation, minimum, maximum, frequencies, percentages) characterized patient demographics, tooth distribution, and occlusal contacts. Given that the data consisted of count data (number of contact points), binary data (presence/absence), nominal data (tooth type, cusp type, side, gender), and paired measurements, which do not assume normality, non-parametric tests were appropriately selected. Therefore, formal normality testing (e.g., Shapiro-Wilk) was not required. Point-level agreement (n=412 contacts) employed confusion matrices (true positive (TP), false negative (FN), false positive (FP), true negative (TN)) to compute Cohen's kappa and diagnostic metrics (sensitivity, specificity, positive predictive value (PPV), negative predictive value (NPV), accuracy). Tooth-level analysis (n=140 teeth) used Wilcoxon signed-rank test for paired contact counts (AP_Total vs. IOS_Total). Premolar vs. molar differences were assessed using chi-square/Fisher's exact tests, the Mann-Whitney U test, Spearman's rank correlation for sensitivity by contact number, and receiver operating characteristic (ROC) curve analysis (area under the curve (AUC)). Agreement patterns (match/under-recording/over-recording) were analyzed by tooth type using chi-square tests. Significance level was set at α=0.05 (two-sided).

## Results

Participant characteristics

Thirty-five patients (11 men, 24 women; mean age 24.6±2.3 years, range 21-29 years) contributed 140 teeth (70 premolars, 70 molars) from one arch side per patient. A total of 412 occlusal contact points were evaluated (Table 1).

**Table 1:** Study sample characteristics

Characteristic	Value
Patients, n	35
- Males	11 (31.4%)
- Females	24 (68.6%)
Teeth, n	140
- Premolars	70 (50%)
- Molars	70 (50%)
Contact points, n	412

Overall diagnostic performance

The point-level confusion matrix across 412 contact points showed 366 true positives (TP), 39 false negatives (FN), 6 false positives (FP), and 1 true negative (TN). Cohen's kappa indicated negligible agreement between the IOS and articulating paper beyond chance (κ=0.014, p=0.680). The scanner demonstrated high sensitivity (90.4%, 95% CI: 87.4-93.0) and high PPV (98.4%, 95% CI: 96.7-99.4), indicating good ability to detect existing occlusal contacts. In contrast, specificity (14.3%, 95% CI: 0.4-57.9) and NPV (2.5%, 95% CI: 0.1-13.2) were low, reflecting limited capability in correctly identifying contact-free areas. Overall accuracy was 89.1% (95% CI: 85.8-92.0; Table 2).

**Table 2:** Diagnostic performance of the Cameo Elegant 3 intraoral scanner compared to 8-μm articulating paper (point-level analysis, n=412 contacts). TP: True positive (contacts correctly identified by both methods); FN: false negative (contacts detected by articulating paper but missed by the scanner); FP: false positive (contacts detected by the scanner but not marked by articulating paper); TN: true negative (areas correctly identified as non-contact by both methods); PPV: positive predictive value; NPV: negative predictive value; CI: 95% confidence interval; κ: Cohen’s kappa coefficient (measure of agreement beyond chance).

Measure	Value (95% CI)	TP	FN	FP	TN
Sensitivity	90.4% (87.4–93.0)	366	39	-	-
Specificity	14.3% (0.4–57.9)	-	-	6	1
PPV	98.4% (96.7–99.4)	366	-	6	-
NPV	2.5% (0.1–13.2)	-	39	-	1
Accuracy	89.1% (85.8–92.0)	367	-	-	-
Cohen's κ	0.014 (p = 0.680)	-	-	-	-

Tooth-level contact number comparison

Articulating paper recorded a mean of 2.89±1.32 contacts per tooth (range 0-8), while the Cameo IOS recorded 2.66±1.38 (range 0-7). The Wilcoxon signed-rank test confirmed a significant difference between the two methods (Z=-4.392, p<0.001), with the scanner tending to under-record contacts in 32 teeth (22.9%) and over-recording in three teeth (2.1%), and numerical equality in 105 teeth (75.0%; Table 3).

**Table 3:** Number of occlusal contacts per tooth recorded by 8-μm articulating paper and the Cameo Elegant 3 intraoral scanner (n=140 teeth) SD=Standard deviation; Z=Wilcoxon signed-rank test statistic.

Parameter	Articulating Paper	Intraoral Scanner	Z value	P value
Contacts per tooth, mean±SD (range)	2.89±1.32(0-8)	2.66±1.38(0-7)	-4.392	< .001
Under-recording	-	32 teeth (22.9%)	-	-
Over-recording	-	3 teeth (2.1%)	-	-

Performance by tooth type (premolars vs. molars)

Premolars (n=160 contact points) demonstrated superior performance with perfect specificity (100%) and PPV (100%), and statistically significant agreement (Cohen κ=0.115, p=0.002). In contrast, molars (n = 252 contact points) showed 0% specificity, negative Cohen kappa (-0.040, p=0.411), and six false positives. Sensitivity was comparable (91.2% premolars vs 89.8% molars), with no significant difference (Mann-Whitney U=2345.00, Z=-0.396, p=0.692). Accuracy was higher for premolars (91.3%) than molars (87.7%; Table 4).

**Table 4:** Diagnostic accuracy of intraoral scanner occlusal contact detection by tooth type TP: true positive; FN: false negative; FP: false positive; TN: true negative; PPV: positive predictive value; NPV: negative predictive value; CI: confidence interval; κ: Cohen’s kappa

Measure	Premolars (n=160)	Molars (n=252)
Confusion Matrix	TP=145, FN=14, FP=0, TN=1	TP=221, FN=25, FP=6, TN=0
Sensitivity (95% CI)	91.2% (85.5–95.5)	89.8% (85.4–93.3)
Specificity (95% CI)	100% (29.2–100)	0% (0–33.6)
PPV (95% CI)	100% (96.8–100)	97.4% (94.4–99.0)
NPV (95% CI)	6.7% (0.2–32.0)	0% (0–12.8)
Accuracy (95% CI)	91.3% (86.0–95.3)	87.7% (83.0–91.5)
Cohen's κ	0.115	-0.040
κ p-value	0.002	0.411

ROC curve analysis

**Figure 4:**
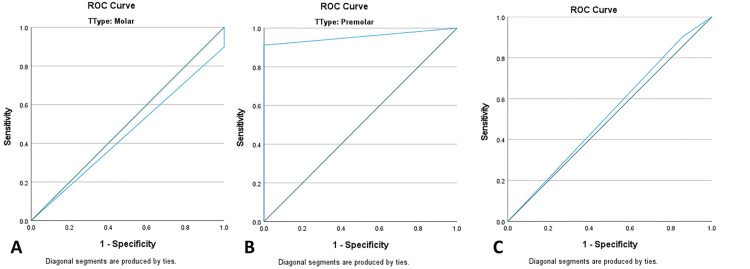
ROC curves comparing intraoral scanner performance: A: Molars (AUC = 0.449) and B: Premolars (AUC = 0.956) and C: Overall (AUC = 0.523) Reference line (AUC = 0.5, dashed)

ROC curve analysis showed excellent discrimination for premolars (AUC=0.956, 95% CI: 0.897-1.000), but poor performance for molars (AUC=0.449, 95% CI: 0.238-0.661). The overall AUC was 0.523 (p=0.833). At the optimal cutoff (0.50), sensitivity remained 90.4% while specificity was 14.3% (Figure 4).

Agreement patterns

Agreement pattern analysis revealed a match in 100 teeth (71.4%), systematic under-recording in 35 teeth (25.0%), and over-recording in three teeth (2.1%). Chi-square testing confirmed significant differences in agreement patterns (χ²=18.234, df=3, p=0.001; Table 5).

**Table 5:** Agreement patterns between articulating paper and intraoral scanner by tooth (n=140) AP: articulating paper; IOS: intraoral scanner; SD: standard deviation; χ²: chi-square; df: degrees of freedom. “AP contacts/tooth” denotes the number of occlusal contact points per tooth recorded by articulating paper. Agreement patterns were defined as follows: “Match”=identical number of occlusal contacts for a given tooth on IOS and AP; “Under-recording”=fewer contacts on IOS than on AP; “Over-recording”=more contacts on IOS than on AP; “Over- and under-recording”=the same tooth exhibiting both extra IOS contacts and missing IOS contacts compared with AP at different sites.

Pattern	N	%	AP contacts/tooth (mean ± SD)
Match	100	71.4	2.50±1.1
Underscore	35	25.0	3.80±1.3
Overscore	3	2.1	1.33±0.6
Over+Under	2	1.4	4.50±1.5
Total	140	100	

## Discussion

Although IOSs have demonstrated clinically acceptable trueness and precision in capturing dental surfaces, as reported in the previous studies [6], this geometric accuracy does not necessarily translate into reliable detection of occlusal contact points. The present study highlights this discrepancy, showing high sensitivity (90.4%) but very low specificity (14.3%) in occlusal contact detection when compared with 8-μm articulating paper.

The IOS exhibited a strong ability to detect true occlusal contacts, as reflected by a high PPV (PPV=98.4%). However, its limited ability to correctly identify non-contact areas resulted in poor specificity and a low NPV. The negligible overall agreement (Cohen’s κ=0.014, p=0.680), despite high sensitivity, can largely be attributed to the extremely low number of true negatives, which significantly affects agreement statistics. Similarly, the high overall accuracy (89.1%) should be interpreted with caution, as it is largely influenced by the high prevalence of true positives.

At the tooth level, the Wilcoxon signed-rank test revealed a statistically significant difference in the number of recorded contacts (p<0.001), with the IOS generally recording fewer occlusal contacts compared with articulating paper (2.66 vs. 2.89 contacts per tooth). Approximately 23% of teeth showed under-recording of occlusal contacts by the scanner, whereas only a small proportion (2.1%) demonstrated over-recording. The majority of teeth (75%) exhibited agreement in the number of contacts, indicating that overall agreement was common despite a tendency toward under-recording.

These findings are consistent with previous studies reporting variability in digital occlusal contact detection. For instance, Manziuc et al. observed that paper-based articulating devices detected more contacts than digital devices, particularly in the posterior regions [9]. Similarly, Esposito et al. reported inconsistent agreement between IOS and articulating paper [13], while Buduru et al. highlighted discrepancies in both the number and location of contacts, especially posteriorly [14]. Such differences across studies may be attributed to variations in study design, scanning protocols, evaluation criteria, or the anatomical complexity of the evaluated regions.

The observed tendency toward under-recording appears to result from several factors. First, anatomical complexity plays a key role, as posterior teeth exhibit deep fissures and complex cusp-fossa relationships that may limit accurate surface capture during scanning [11]. Second, minor inaccuracies during bite registration and virtual model alignment may introduce discrepancies in contact detection. Third, the limitations of software-based collision detection algorithms, along with the absence of dynamic functional integration, may limit IOS's ability to fully replicate the physical occlusal interactions recorded by thin articulating paper [15]. Finally, intraoral conditions, such as the presence of saliva and slight mandibular movement during scanning, may result in missed contacts. These factors align with systematic reviews highlighting the importance of dynamic validation in improving the accuracy of digital occlusal records [16]. Tooth-type analysis revealed that premolars demonstrated significantly better agreement with articulating paper compared with molars. Premolars showed statistically significant agreement and better discriminatory ability (κ=0.115, p=0.002), whereas molars exhibited negligible agreement and poor diagnostic performance (κ=-0.040, p=0.411). This difference is likely related to anatomical factors. Premolars have simpler occlusal morphology and smaller surface areas, facilitating a more accurate digital capture. In contrast, molars present broader occlusal tables, multiple cusps, and more complex contact relationships, increasing the likelihood of scanning and alignment errors. This anterior-posterior gradient in accuracy is consistent with in vitro findings showing that intraoral scanner precision decreases from anterior to posterior regions due to arch distortion and increasing anatomical complexity [11]. ROC curve analysis further supported these findings, showing excellent discriminatory ability in premolars (AUC=0.956, 95% CI: 0.897-1.000) and poor performance in molars (AUC=0.449, 95% CI: 0.238-0.661). This discrepancy likely reflects the strong influence of occlusal morphology on digital detection accuracy rather than an inherent limitation of the scanner itself.

Despite these limitations, the IOS demonstrated acceptable performance in detecting occlusal contacts in premolar regions, supporting its potential use as an adjunctive diagnostic tool. However, its very low specificity and reduced reliability in molar regions indicate that it should not be relied upon as a standalone method for occlusal assessment.

From a clinical perspective, these results suggest that digitally detected occlusal contacts should be interpreted with caution, particularly in posterior regions. Clinicians are advised to verify scanner findings with conventional 8-μm articulating paper to ensure accurate decision-making, especially during prosthodontic rehabilitation and restorative procedures that require precise occlusal adjustment [13]. While the scanner may serve as a useful adjunctive tool in areas with simpler anatomy, such as premolars, greater caution is advised when assessing molars due to their complex morphology [11]. Future integration with dynamic technologies, such as jaw-tracking systems, may further enhance the clinical utility of intraoral scanners [15].

Limitations

This study has several limitations. First, the evaluation of a single intraoral scanner (Cameo Elegant 3) limits the generalizability to other systems with varying hardware/software. Second, the analysis was restricted to static contacts at maximum intercuspation without evaluating dynamic occlusion. Third, the small sample size (n=35; ages 21-29 years) may not be representative of the broader population. In addition, although the digital alignment protocol relied on software-assisted automation to reduce human variance, the study is limited by its single-examiner design. The absence of formal test-retest repeatability assessments, including intra- and inter-examiner reliability analyses (such as Cohen’s kappa or intraclass correlation coefficients) and repeat scan reproducibility evaluations, represents a methodological limitation. Future studies evaluating intraoral scanner systems should incorporate dedicated reproducibility and multi-examiner validation protocols. Finally, the limited number of true negatives (n=1) affected specificity calculations and agreement measures, particularly Cohen’s kappa. Future studies should include multiple scanners, larger and more diverse samples, dynamic occlusal analysis, and multi-examiner validation.

## Conclusions

This study demonstrated that the Cameo Elegant 3 IOS exhibited high sensitivity (90.4%) but very low specificity (14.3%) in detecting static occlusal contact points compared to 8-μm articulating paper, resulting in significant under-recording, especially in molar regions. While the scanner showed acceptable performance in premolars, its overall agreement with the gold standard was negligible (κ=0.014). These findings highlight the current limitations of static intraoral scanning in accurately recording occlusal contacts, especially in posterior teeth with complex morphology.

IOSs should be considered valuable adjunctive tools in digital workflows rather than complete replacements for conventional occlusal assessment. Combining digital scanning with thin articulating paper remains essential for reliable occlusal evaluation in clinical practice. Future research should evaluate multiple scanner systems, incorporate dynamic occlusal analysis, and include broader populations to better define the role of intraoral scanners in precise occlusal diagnosis.
